# Early latency evoked potentials can no longer be considered an infallible predictor of neurologic outcome

**DOI:** 10.1186/s13054-020-03056-5

**Published:** 2020-06-09

**Authors:** Patrick M. Honore, Leonel Barreto Gutierrez, Luc Kugener, Sebastien Redant, Rachid Attou, Andrea Gallerani, David De Bels

**Affiliations:** 1 ICU Department, Centre Hospitalier Universitaire Brugmann-Brugmann University Hospital, Place Van Gehuchtenplein, 4, 1020 Brussels, Belgium; 2 Centre Hospitalier Universitaire Brugmann, Brussels, Belgium

We read with great interest the recent article by Rothstein et al. who conclude that the absence of the SSEP N20 cortical wave remains one of the most reliable early prognostic tools for identifying unfavorable outcome in the patients with severe anoxic-ischemic encephalopathy, whether or not they have been treated with therapeutic hypothermia [1]. The reliability of SSEP as a predictor of poor outcome in the era of therapeutic hypothermia following cardiac arrest has been questioned [2]. A systematic review of the literature identified cases in which there was a good outcome despite bilateral absence of the cortical SSEP N20 response and concluded that SSEP can no longer be considered an infallible predictor of neurologic outcome [2]. Early latency evoked potentials may not always be the best tool to identify the prognosis of a coma after cardiac failure [3]. Middle latency evoked potentials are elicited by cortico-cortical projections and appear up to 100 ms following stimulation of the median nerve [3]. They are believed to better reflect functional connectivity and thus allow prognostication of awakening, but due to challenging technical and practical issues, they are used far less commonly than early latency potentials. An Austrian study before the therapeutic temperature management (TTM) era reported a positive predictive value for awakening as high as 97% [3]. Long-latency evoked potentials represent higher-order cortical reactions occurring more than 100 ms after stimulation of the primary sensory areas. It is a common practice, especially in comatose patients, to record these with auditory stimulations; response to deviant stimuli in a sequence of standard stimuli, also called “mismatch negativity,” has been described several years ago, in the pre-TTM era, to be strongly associated with awakening after a latency of several days to weeks following cardiac arrest [4]. More recently, this approach has been developed and refined though an automated voltage topography analysis of recordings performed during acute post-cardiac arrest coma, on a case-by-case basis: progression of auditory discriminations between the first and the second assessments (both recorded within the first 48 h after the arrival of the patient to the hospital) heralded good recovery for the vast majority of patients [5]. In the near future, middle and late latency evoked potentials and mismatch negativity may replace early latency SSEP and may provide much more accurate prognostication in patients in coma after cardiac arrest.

## Data Availability

Not applicable.

## Abbreviations

SSEP: Somatosensory evoked potentials
TTM: Therapeutic temperature management
